# Liver Transplantation Outcomes in Younger Versus Older Adult Recipients: The Edmonton Experience

**DOI:** 10.1155/joot/8889823

**Published:** 2025-11-17

**Authors:** U. Mathuram Thiyagarajan, B. Marfil-Garza, Khaled Dajani, Blair Anderson, David Bigam, Aldo Montano-Loza, A. M. James Shapiro

**Affiliations:** Department of Surgery, Medicine and Surgical Oncology, University of Alberta Hospital, Edmonton T6G 2B7, Canada

## Abstract

**Introduction:**

Liver transplantation (LT) offers a lifesaving treatment for patients with end-stage liver disease (ESLD). There have been conflicting reports of outcomes in younger and elderly patients undergoing LT. This study assesses the outcomes of younger and elderly LT recipients by complications and graft survival at early and late time-points, up to 10 years.

**Patients and Methods:**

This retrospective study was conducted on a prospectively collected database of patients who underwent LT between January 2011 and December 2021 at the University of Alberta Hospital in Edmonton, Canada.

**Results:**

A total of 696 patients who were 18 years and older were included and then classified into two groups: the younger adult group (YG; *n* = 631, < 65 years old) and the older adult group (OG; *n* = 65, > 65 years old). The YG was sicker, with a high model for ESLD (MELD) score, while the OG had a high incidence of coronary artery disease (CAD), hypertension, smoking, and hepatocellular carcinoma. The YG had a higher incidence of postoperative pleural effusion requiring drainage (108/631 [17%] versus 4/65 [6%]; *p* value < 0.02) and more rejection episodes (202/631 [32%] versus 10/65 [15%]; *p* value < 0.04). However, the OG had more hepatic artery thrombosis (HAT) (4/65 [6.1%] versus 10/631 [1.6%]; *p* value 0.03). CAD and smoking history were associated with lower patient and graft survivals; acute rejection episodes were also associated with significantly lower graft survival.

**Conclusion:**

The patient and graft survival between the YG and OG are comparable at 30 days, 90 days, 1, 5 and 10 years. A history of CAD, smoking and rejection episodes decreased graft survival and age alone should not be a contraindication for LT.

## 1. Introduction

Starzl et al. first demonstrated the clinical feasibility of a successful liver transplantation (LT) in 1963, though earlier outcomes were poor [1, 2]. Substantial morbidity and mortality in the early experience reflected poor-quality liver donors, lack of preservation solutions and ineffective and risky immunosuppression [3, 4]. However, with the advent of cyclosporine and then tacrolimus at the later part of the 20th century and made the LT a highly effective treatment option for patients with end-stage liver disease (ESLD) [5, 6].

The world population is increasing, and thus there is a proportionate growth in the older adult population [7, 8]. More specifically, the growth of the elderly adult population is currently burgeoning [8]. It is also predicted that by 2030, 1 in 6 people will be aged 60 years or over 1.4 billion then to double 2.1 billion in 2050 [7]. Some developed nations like Japan already have 30% of the population > 60 years old. In the United States, the population > age 65 years old was 13% (39.6 million) in 2009 and is expected to reach 19% (72 million) in 2030 [9]. Moreover, low- and middle-income countries will have two-thirds of the world's population over 60 years [7]. Although there has been a heterogeneity of defining the “older adult person,” conventional definition of people > age 65 years old has been accepted in most clinical research [10–12]. Hence, in this study we define the older adult recipients who are > 65 years old at the time of undergoing a LT for ESLD.

The liver is capable of remarkable regeneration even after 75% of its volume has been removed in a healthy adult [13]. Ageing is a process in which the individual spontaneously loses the capability of maintaining the homeostasis secondary to either a structural alteration or due to a dysfunction, thereby becoming vulnerable to an external insult [14]. Liver disease in the older adult population is underdiagnosed and there is a higher risk of progression to fibrosis in hepatitis C infection, alcoholic hepatitis than in their younger counterparts [14–16]. Nonetheless, the exact mechanisms for this predisposition are not well understood. The incidence of nonalcoholic fatty liver disease (NAFLD) is also higher in the older adult population [17, 18]. Therefore, the older adult recipients have more predisposition for advanced liver disease progressing to ESLD, and this unfortunate cohort needs closer attention. As the LT offers a highly effective life-saving treatment for ESLD, further research needs to focus specifically on the outcomes in older adult recipients.

### 1.1. Definition of Terms

#### 1.1.1. Orthotopic LT

A whole or partial liver graft is taken one from individual from the same species and transplanted into the same site after the diseased liver has been removed.

#### 1.1.2. Model for ESLD (MELD)

The model for ESLD, or MELD, is a scoring system for assessing the severity of chronic liver disease. It was initially developed to predict mortality within 3 months of surgery in patients who had undergone a transjugular intrahepatic portosystemic shunt (TIPS) procedure, and was subsequently found to be useful in determining prognosis and prioritizing for receipt of a liver transplant.

#### 1.1.3. Status of Transplant Recipient

Status 1—at home, status 2—admitted to hospital, status 3—in intensive care unit (ICU) not intubated, status 4—intubated in ICU, F—denotes fulminant liver failure.

#### 1.1.4. Acute Rejection Episodes

Although acute rejection episodes commonly occur within 90 days, no specific chronological timeframe was defined in our study. All reported episodes of acute rejection were confirmed by histopathological examination of graft biopsy.

## 2. Methods

All adult LTs performed at the University of Alberta Hospital over 11 years (January 2011 to December 2021) were included in this study. This study was approved by the Institutional Review Board of the University of Alberta Hospital (approval reference Pro00102861). Because this was a retrospective study utilizing existing clinical data, the requirement for individual patient informed consent was formally waived by the Board. There was no conflict of interest among any of the authors. Relevant parameters were retrieved from a prospectively collected comprehensive database. The inclusion criterion was all patients aged 18 years or older who received a liver transplant. Recipients were classified into two groups: the younger adult group (YG–< 65 years old) and the older adult group (OG–≥ 65 years old) before data analysis. All patients were followed up by the local or regional LT and hepatology team, unless the patients expressed a different preference or emigrated to other countries.

Demographic parameters, including weight, body mass index (BMI), obesity, hypertension, diabetes, smoking history, hyperlipidemia, alcohol abuse, primary diagnosis for ESLD, HCV infection, hepatocellular carcinoma (HCC), and the type of liver graft, were included in the statistical analysis.

Primary parameters for analysis included patient and graft survival at 30 days, 90 days, 1, 3, 5, and 10 years. Secondary outcomes included reoperation within 90 days, length of hospital stay, significant postoperative biliary, vascular, respiratory, cardiac, surgical complications (Clavien-Dindo 3 or more) [19] and rejection episodes occurring at any time-point post-transplant.

### 2.1. Patient Evaluation and Immunosuppression Protocol

All potential liver transplant recipients were evaluated in a comprehensive multidisciplinary transplant clinic after the hepatology team established decompensated ESLD. Relevant blood investigations included a complete blood count, clotting profile, liver function tests, urea, electrolytes, random glucose, total protein, creatinine, blood group/typing, and relevant serological tests for active or previous viral infections. Radiological investigations included an abdominal ultrasonogram, triphasic abdominal computerized tomography (CT scan), or magnetic resonance imaging (MRI) of the abdomen, as appropriate. Patients over 50 years or with a significant smoking history underwent pulmonary function tests and arterial blood gas analysis. Cardiological evaluation consisted of an electrocardiogram (ECG) and echocardiography. Those at high risk for coronary artery disease (CAD) or with a previous history of CAD underwent cardiac positron emission tomography (PET) and cardiac angiogram, as appropriate. Patients with positive findings were seen by appropriate specialists, and transplant anesthesia consultants provided final clearance. Specialist dieticians assessed nutritional status, while social workers further explored the patient's support system, addictions, and provided feedback on areas of improvement or concern for transplant candidacy, compliance, and recidivism risk. In line with local practice, all liver transplant candidates, excluding super-urgent cases, were reviewed in a liver transplant multidisciplinary meeting (MDT) before active listing.

Induction immunosuppression included basiliximab (Simulect, Novartis Pharmaceuticals Canada Inc., Quebec, Canada) at a dose of 20 mg given intravenously (IV) in the operating room and on the 4th postoperative day. Early post-transplantation immunosuppression was achieved with tacrolimus, administered orally or through a nasogastric tube at a dose of 0.025 mg/kg every 12 h, usually initiated 3 days after LT. Mycophenolate mofetil was added at a dose of 1000 mg, administered orally or through the nasogastric tube every 12 h.

Patients with acute kidney injury/renal failure or renal impairment in the pretransplant period or after LT were treated with calcineurin-free sirolimus at a dose of 0.1 mg/kg once daily, together with mycophenolate mofetil. Corticosteroid use was avoided in most recipients, except those with baseline autoimmune hepatitis, and in that setting, it was used sparingly. Therapeutic levels of tacrolimus and sirolimus were maintained at 8–10 μg/L and 7–10 μg/L, respectively. Antimicrobial prophylaxis was provided by meropenem 500 mg every 6 h IV and metronidazole 500 mg every 8 h, beginning intraoperatively and continuing for 24 h. Patients at high risk for vascular complications (previous portal vein thrombosis, complex arterial construction) received IV heparin at a rate of 300 IU/hour, then increased to 500 IU/hour as appropriate. After a stable postoperative course, anticoagulation was converted to acetylsalicylic acid 81 mg daily (for those with complex arterial reconstruction, aortic conduit, or those who received sirolimus) or warfarin (for portal vein thrombosis).

### 2.2. Statistical Analysis

The data are presented as mean ± standard deviation (SD), and statistical analysis was performed using Instat software for Windows (GraphPad, San Diego, USA). Normality testing of data was conducted using the Kolmogorov–Smirnov test. Continuous variables were compared using the Student's *t*-test or the Mann–Whitney *U*-test as appropriate, and categorical variables were analyzed using Fisher's exact or chi-square test. A significance level of *p* < 0.05 was defined in our analysis. Kaplan–Meier survival curves were generated using log-rank statistics to assess patient survival between the two cohorts. Patient and graft survival at 30 days, 90 days, 1 year, 5 years, and 10 years were extracted from the survival curves.

## 3. Results

There were a total of 696 patients in our study population, with 631 in the YG–recipients < 65 years old and the remaining 65 patients in the OG–recipients > 65 years old. The YG and OG had comparable demographic parameters, including sex, weight, BMI, obesity, diabetes, hyperlipidemia, and alcohol abuse history.

The OG included more patients (15/65, 23%) with CAD, hypertension (36/65, 55%), and a smoking history (47/65, 72%). While looking at the etiology of ESLD, the YG had a significantly higher incidence of alcoholic liver disease (ALD) (Table 1–99/631, 16%) and primary sclerosing cholangitis (PSC) (Table 1–74/631, 12%). However, the OG had a higher number of patients with HCC (Table 1–32/65, 49%) as the primary indication for LT.

Interestingly, more YG patients had hepatorenal syndrome (120/631, 19% vs. 3/65, 5%; *p* value < 0.001, Table 1), but there was a similar incidence of hepatopulmonary syndrome (14/631, 2% vs. 3/65, 4%; *p* value 0.21, Table 1). The YG patients had significantly higher MELD-Na scores (26, IQR 20–30.5 vs. 14, IQR 9–19.5; *p* value < 0.001; Table 1) compared to the OG. Notably, serum bilirubin (58, IQR 28–170 vs. 32, IQR 18.5–56.5; *p* value < 0.002; Table 1), creatinine (78, IQR 61–105 vs. 81, IQR 63.5–94; *p* value < 0.0001; Table 1), and INR (1.4, IQR 1.2–1.9 vs. 1.2, IQR 1.1–1.5; *p* value < 0.01; Table 1) were higher in the OG. The YG also had more patients needing intraoperative hemodialysis (87/631, 14% vs. 2/65, 3%; *p* value < 0.01; Table 1). Moreover, pretransplant hemodialysis was more frequent in the YG than OG (70/631, 11% vs. 2/65, 3%; *p* value < 0.06; Table 1), although it did not reach statistical significance.

Although the majority of recipients received donation after brain stem death (DBD) liver grafts in both groups, a higher proportion of patients in the YG underwent live donor LT (77/63, 13% vs. 2/65, 3%; *p* value < 0.02; Table 1). The length of hospital stay was similar in the YG and OG.

### 3.1. LT and Postoperative Complications

All postoperative complications were included; regardless of the time elapsed since LT. Operative time (6.5, IQR 4.8–6.8 vs. 6.1, IQR 4.5–6.2 h; *p* value 0.11, Table 2), blood transfusion (3.3, IQR 3–4.9 vs. 2.6, IQR 2.3–3.7 units; *p* value 0.29, Table 2), and days in the ICU (8, IQR 6–9 vs. 8, IQR 6–9.2 days; *p* value 0.98, Table 2) were comparable between groups. Surprisingly, more patients from the YG suffered respiratory failure (123/631 [19%] vs. 2/65 [3%]; *p* value < 0.0003, Table 2) during ICU stay. Pleural effusions requiring intervention were more common in the YG (108/631 [17%] vs. 4/65 [6%]; *p* value < 0.002) than in the OG. Patients requiring hemodialysis after LT, primary nonfunction (PNF) of the liver, and biliary complications were comparable in the YG and OG (Table 2).

However, a higher proportion of patients in the OG developed hepatic artery thrombosis (HAT) (4/65, 6% vs. 10/631, 1.6%; *p* value < 0.03, Table 2) than in the YG. Other vascular complications, including hepatic artery stenosis, portal vein thrombosis, portal vein stenosis, and inferior vena cava (IVC) occlusion, were similar among the groups. Moreover, the incidence of cardiac complications, systemic infections, and reoperation after LT were comparable between the YG and OG (Table 2).

The YG patients had double the risk of biopsy-proven acute rejection than the OG (202/631, 32% vs. 10/65, 15%; *p* value < 0.004, Table 2). The majority of these episodes were steroid-sensitive or managed with increasing immunosuppression (conservatively treated), and only 2% of the transplanted patients (15 of the 696) suffered steroid-resistant rejection requiring further treatment with antithymocyte globulin or anti-CD52 monoclonal antibody.

### 3.2. Patient and Graft Survival

Analysis of mortality was performed using a Cox proportional hazards model to assess the impact of age on patient survival (hazard ratio–0.86 [0.45–1.66, *p*=0.654] Table 3) and graft survival (hazard ratio–0.87 [0.48–1.58, *p*=0.641] Table 3), and no difference was found. Further analysis showed that patient sex (hazard ratio–0.95 [0.65–1.39, *p*=0.784], Table 3), HCC [hazard ratio–1.42 (0.96–2.10, *p*=0.076), Table 3], and MELD-Na score (hazard ratio–1.02 [1.00–1.03, *p*=0.087], Table 3) also did not impact patient survival.

However, patients with CAD (hazard ratio–1.87 [1.18–2.94, *p*=0.007] and smoking (hazard ratio–1.47 [1.01–2.14, *p*=0.044], Table 3) had poor survival. Similarly, patients' sex, HCC, and MELD-Na score did not impact graft survival, but CAD (hazard ratio–1.69 [1.01–2.85, *p*=0.048], Table 3), smoking (hazard ratio–1.54 [1.03–2.28, *p*=0.032]), and acute rejection episode (hazard ratio–2.19 [1.42–3.36, *p* < 0.0001]) were associated with lower graft survival.

**Figure 1:**
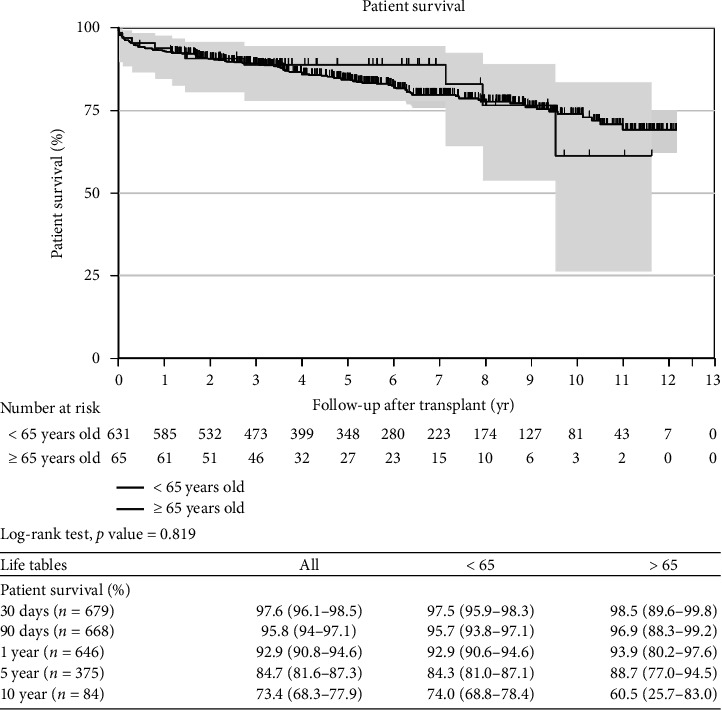
Patient survival–unadjusted (Kaplan–Meir curves).

**Figure 2:**
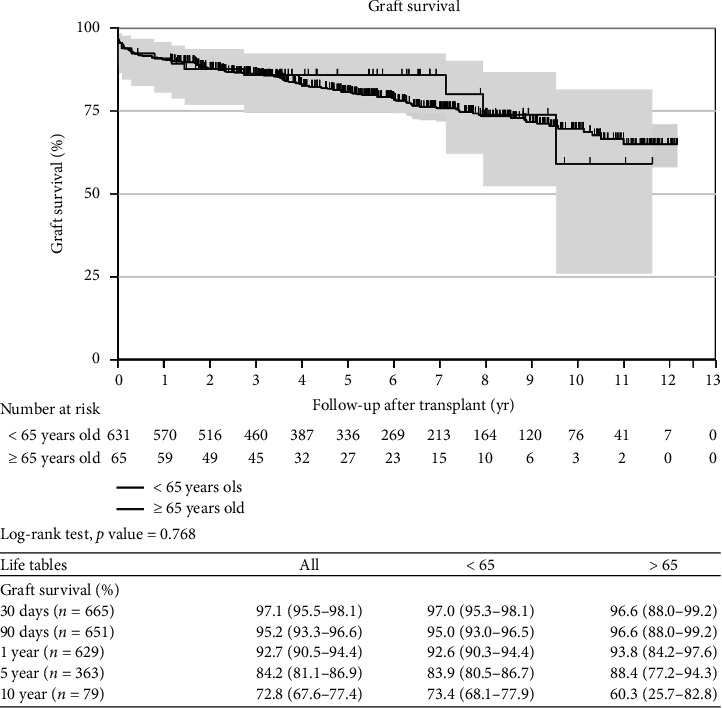
Graft survival–unadjusted (Kaplan–Meir curves).

**Figure 3:**
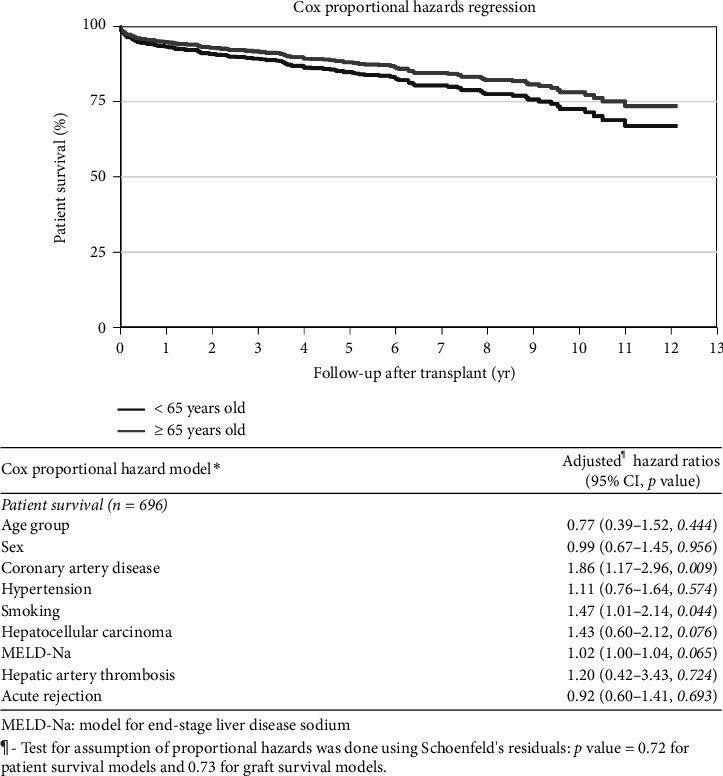
Patient survival–adjusted (for age, sex, CAD, hypertension, smoking HCC, MELD-Na score, HAT and acute rejection).

**Figure 4:**
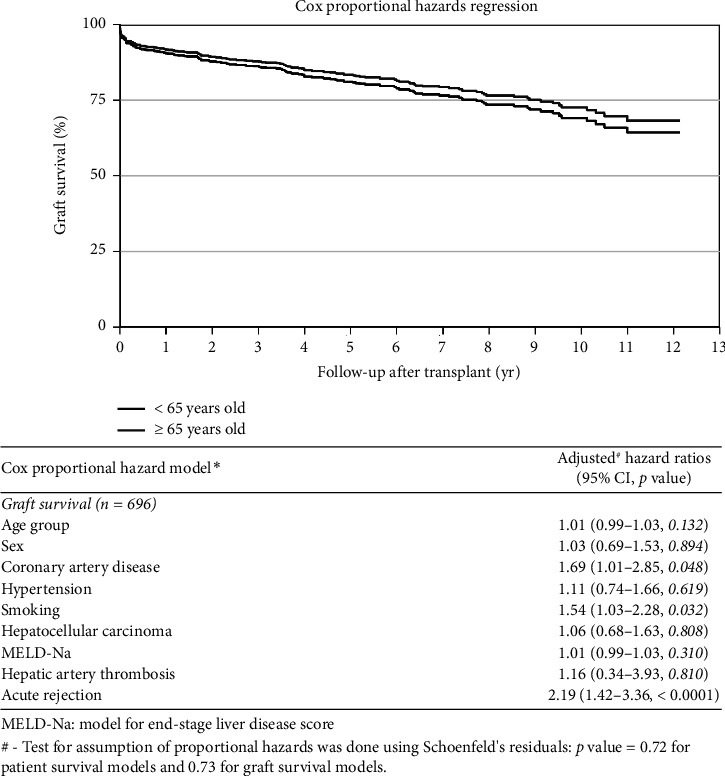
Graft survival–(adjusted age, sex, CAD, hypertension, smoking HCC, MELD-Na score, HAT, and acute rejection).

Kaplan–Meier curves and log-rank tests were used to assess mortality at 30, 90 days, 1, 5, and 10 years and found comparable survival between the YG and OG (Figures 1 and 2). These findings remain unchanged even after adjusting for age, sex, HCC, and MELD-Na score (Figures 3 and 4). Overall patient survival at 5 and 10 years was 92.9%, 84.7%, and 73.4%, respectively. Correspondingly, overall graft survival at 5 and 10 years was 92.7%, 84.2%, and 72.8%, respectively.

## 4. Discussion

We herein provide a retrospective comparison of a prospective cohort of older and younger patients undergoing LT with respect to outcome and complications. Our collective experience suggests that outcomes are both comparable and excellent in highly selected older adult patients being considered for LT compared to younger adult patients, and chronological age alone should not be used as a prohibitive bar provided other comorbidities are low.

In our study, younger adult patients were sicker with higher MELD-Na scores, although there was no major difference in the etiology of ESLD except alcoholic cirrhosis and PSC. There was also a higher incidence of post-transplant respiratory failure after LT and required longer period of ventilator support than in the elderly group and likely from a higher MELD-Na score as reported previously by others [18, 19].

Understandably, the OG had higher rates of hypertension, smoking history, and CAD, which were associated with less long-term outcome survival but short-term outcomes within the first 5 years post-transplant were excellent. Previous research showed an incidence of CAD as 18%–27% in patients with ESLD which is higher than that seen in the general population [20–24]. Alexander et al. reported that the combination of smoking, hypertension and CAD was associated with major cardiac adverse events after transplantation [23]. Our study population had 13% of CAD combined; of course, the major share went with OG, reaching 23%. Interestingly, the OG had significantly less perioperative cardiac events despite more patients with a history of CAD. Similarly, low respiratory complications and less time spent in the ICU was observed in the OG, likely reflecting a far more stringent patient selection practice for patients who are > 65 years old together with preoperative optimization. This was highlighted in previous studies suggesting that age alone should not be a contraindication for LT as long as the pretransplant cardiovascular fitness and respiratory optimization have been established [25–27].

In regard to the type of the graft, YG received high percentage of live donor (13%) compared to the elderly group (3%); however, donation after circulatory death (DCD) livers were numerically higher but not significantly so (15% OG vs. 10%, *p*=0.19). The International LT Society recommends recipients should be < 60 years old for DCD LT [28]. In the OG, none developed PNF, and there was comparable intraoperative coagulopathy evident by similar blood transfusion requirements. Similar rates of biliary complications were observed between groups. Thus, at least in our local experience, highly selected elderly patients may safely receive a DCD liver with favorable donor characteristics including age, cold ischemia time, and quality.

The OG had a higher incidence of HAT (6%) when compared to YG (1.6%), making the overall incidence 2% in our study population which is less than the reported incidence [29–31]. Approximately half of the OG patients were transplanted for HCC with MELD-Na exception advantage and were immunosuppressed with sirolimus-based (and usually calcineurin-inhibitor free) immunosuppression in line with our local protocols. Perhaps sirolimus contributed to this higher rate of HAT, but more likely advanced age with increasing severity of atherosclerosis was the major factor.

Notably, there was a high incidence of pleural effusion requiring drainage in the YG, possibly related to high MELD-Na and high rate of respiratory failure following the LT. Similar findings have been reported by other studies too [29–33]. We do not have a data on the incidence of ascites prior to LT which is a well-described risk factor for postoperative pleural effusions [32, 34]. Moreover, this is a unique finding that requires further investigation as pleural effusion following LT is associated with significantly high morbidity [32–35].

Cumulative rates of acute rejection episodes in our study were documented throughout the follow-up period. As anticipated, the YG had a higher rate of rejection (32%) compared to OG (15%, *p*=0.004). Previous studies suggest that elderly patients have better medication adherence compared to their younger counterparts [36] and may partly explain these findings, but younger patients with more robust immunologic responses may also explain the higher rate of rejection, and a likely immunological senescence in older recipients has been associated with less risk of rejection in LT [37].

A major strength of this study is that comprehensive data were collected prospectively after LT by a dedicated research team. Secondly, all the patients were contacted regularly until their death, with low rates of patients lost to follow-up. However, there are inherent weaknesses, especially the retrospective analysis and the stringent bias in selecting only stable older adult recipients with low comorbidities for inclusion. Another limitation is that donor age data were not available in our dataset due to institutional privacy safeguards, precluding analysis of the potential impact of donor age on recipient and graft survival. Additionally, we were unable to quantify the presumably high number of patients who were never referred or excluded early in the process. This bias is intended to ensure the appropriate utility of scarce liver organ resources. The main strength of the current study lies in the excellent outcomes achievable with appropriate patient selection. Our bias to include a much higher incidence of patients with HCC and lower MELD-Na scores likely reflects the observed outcomes. The lack of relevant data on frailty scoring, sarcopenia quantification, pretransplant ascites, and rates of hydrothorax would have also provided valuable information.

## 5. Conclusion

Recipients more than 65 years old had comparable outcomes after LT when compared to younger adult recipients, and in highly selected cases outcomes are excellent. While stringent patient selection should occur, chronological age alone should not be seen as an insurmountable barrier to LT.

## Figures and Tables

**Table 1:** Demographic parameters.

	Younger adults (YG)	Older adults (OG)	*p* value
	(*n* = 631)	(*n* = 65)	
Age (years)	55.5 (44.6–60.5)^∗^	66.6 (65.7–67.8)^∗^	**< 0.001**
Sex (male : female)	420 : 211	39 : 26	0.96
Weight (kilograms)	80.4 (68.4–94.5)^∗^	76.6 (66–88.2)^∗^	0.12
Body mass index (BMI–kg/m^2^)	26 (23–30.5)^∗^	25.5 (23.3–29.5)^∗^	0.48
Obesity (BMI > 30)	177/631 (35%)	15/65 (23%)	0.30
Coronary artery disease	75/631 (12%)	15/65 (23%)	**0.018**
Diabetes	164/631 (26%)	20/65 (31%)	0.46
Hypertension	222/631 (35%)	36/65 (55%)	**< 0.001**
Smoking history	372/631 (59%)	47/65 (72%)	**0.04**
Hyperlipidemia	112/631 (18%)	16/65 (25%)	0.11
Alcohol abuse	262/631 (42%)	20/65 (31%)	0.11
Primary diagnosis			
ALD	99/631 (16%)	3/65 (2%)	**< 0.001**
NASH/NAFLS	55/631 (9%)	7/65 (9%)	0.81
HCC	149/631 (23%)	32/65 (49%)	**< 0.001**
HCV cirrhosis	167/631 (26%)	20/65 (31%)	0.46
HBV cirrhosis	11/631 (2%)	2/65 (3%)	0.34
PBC	53/631 (8%)	1/65 (1.5%)	0.15
PSC	74/631 (12%)	0/65 (0%)	**0.006**
Other causes	23/631 (4%)	0/65 (0%)	0.71
HRS	120/631 (19%)	3/65 (4%)	**0.001**
HPS	14/631 (2%)	3/65 (4%)	0.21
Preoperative PVT^†^	120/631 (19%)	19/65 (29%)	**< 0.01**
Preoperative parameters			
MELD-Na	26 (23–30.5)^∗^	14 (9–19.5)^∗^	**< 0.001**
Sodium	136 (133–138)^∗^	137 (134–139)^∗^	0.32
Bilirubin	58 (28–170)^∗^	32 (18.5–56.5)^∗^	**< 0.002**
Creatinine	78 (61–105)^∗^	81 (63.5–94)^∗^	**< 0.001**
INR	1.4 (1.2–1.9)^∗^	1.2 (1.1–1.5)^∗^	**< 0.01**
Intraoperative HD	87/631 (14%)	2/65 (3%)	**< 0.01**
Preop hemodialysis	70/631 (11%)	2/65 (3%)	0.06
Other parameters			
DBD graft	491/631 (77%)	53/65 (82%)	0.63
Live donor graft	77/631 (13%)	2/65 (3%)	**< 0.02**
DCD graft	63/631 (10%)	10/65 (15%)	0.19
Length of hospital stay (days)	18 (12–31)^∗^	21 (12–34.5)^∗^	0.72

*Note:* Bold values indicate statistical significance between the two groups.

^∗^Median and interquartile range (IQR).

^†^PVT-portal vein thrombosis on the main vein or at either branch.

**Table 2:** Intraoperative and postoperative parameters.

	Younger adults (YG)	Older adults (OG)	*p* value
	(*n* = 631)	(*n* = 65)	
Operative time (in hours)	6.5 (4.8–6.8)^∗^	6.1 (4.5–6.2)^∗^	0.11
Blood transfusion (in units)	3.3 (3–4.9)^∗^	2.6 (2.3–3.7)^∗^	0.29
Days in intensive care unit	8 (6–9)^∗^	8 (6–9.2)^∗^	0.98
Patients with respiratory failure	123/631 (21%)	2/65 (3%)	**< 0.002**
Days on ventilator	4.1 (3–9)^∗^	2.5 (2–7)^∗^	0.06
Reintubation	84/631 (13%)	5/65 (7%)	0.24
Tracheostomy	45/631 (7.1)	5/65 (7%)	0.80
Patients on HD after LT	96/631 (15%)	8/65 (12%)	0.71
PNF	13/631 (2%)	0/65 (0%)	0.62
Respiratory complications^††^			
Pleural effusion	108/631 (17%)	4/65 (6%)	**< 0.02**
Hemothorax	2/631 (0.2%)	0/65 (0%)	1.00
Pneumothorax	16/631 (2.5%)	1/65 (1.5%)	1.00
Pulmonary embolism	3/631 (0.5%)	1/65 (1.5%)	0.32
Biliary complications^††^	153/631 (24%)	12/65 (18%)	0.54
Early bile leak	33/631 (5.2%)	1/65 (1.5%)	0.35
Late bile leak	7/631 (1%)	0/65 (0%)	1.0
Biliary obstruction/stricture	110/631 (17%)	11/65 (17%)	1.00
Biliary stones	3/631 (0.5%)	0/65 (0%)	1.00
Vascular complications^††^			
HAT	10/631 (1.6%)	4/65 (6.1%)	**< 0.03**
Hepatic artery stenosis'	35/631 (5.5%)	2/65 (3%)	0.56
Portal vein thrombosis	7/631 (1%)	0/65 (0%)	1.00
Portal vein stenosis	8/631 (1%)	1/65 (1.5%)	0.58
IVC occlusion	7/631 (1%)	0/65 (1%)	1.00
Cardiac complications^††^			
Cardiac arrest	9/631 (1.4%)	1/65 (1.5%)	1.00
Cardiac dysfunction	21/631 (3%)	1/65 (1.5%)	0.28
Cardiac failure	4/631 (0.6%)	4/65 (6%)	0.38
CAD	2/631 (0.3%)	0/65 (0%)	1.00
Myocardial infarction	4/631 (0.6%)	0/65 (0%)	1.00
Pericardial effusion	1/631 (0.1%)	1/65 (1.5%)	0.17
Surgical complications^††^			
Laparotomy for bleeding	64/631 (10%)	7/65 (10%)	0.83
Laparotomy for bile leak	41/631 (6.5%)	8/65 (12%)	0.11
Abdominal collections	24/631 (3.8%)	0/65 (0%)	0.15
Compartment syndrome	8/631 (1.2%)	0/65 (0%)	1.00
Wound dehiscence	8/631 (1.2%)	2/65 (3%)	0.28
All time rejection^§^			
All rejection episodes	202/631 (32%)	10/65 (15%)	**< 0.004**
Steroid sensitive	124/631 (20%)	6/65 (9%)	**< 0.04**
Steroid resistant	15/631 (3%)	0/65 (0%)	0.38
Conservatively treated	63/631 (10%)	4/65 (6%)	0.38
Infection^§^			
Bacterial	90/631 (14%)	4/65 (6%)	0.08
Fungal	5/631 (1%)	0/65 (0%)	1.00
PTLD	11/631 (1.7%)	0/65 (0%)	0.61

*Note:* Bold values indicate statistical significance between the two groups.

^∗^Median and interquartile range (IQR).

^††^Clavien Dindo > 3a grade complication.

^§^Clavien Dindo grade 2 complication.

**Table 3:** Cox proportional hazard model assessing patient and graft survival.

Cox proportional hazard model^∗^	Adjusted hazard ratios^∗^ (95% CI, *p* value)
Patient survival (*n* = 696)	
Age group	0.77 (0.39–1.52, 0.444)
Sex	0.99 (0.67–1.45, 0.956)
Coronary artery disease	1.86 (1.17–2.96, 0.009)
Hypertension	1.11 (0.76–1.64, 0.574)
Smoking	1.47 (1.01–2.14, 0.044)
Hepatocellular carcinoma	1.43 (0.60–2.12, 0.076)
MELD-Na	1.02 (1.00–1.04, 0.065)
Hepatic artery thrombosis	1.20 (0.42–3.43, 0.724)
Acute rejection	0.92 (0.60–1.41, 0.693)
Graft survival (*n* = 696)	
Age group	1.01 (0.99–1.03, 0.132)
Sex	1.03 (0.69–1.53, 0.894)
Coronary artery disease	1.69 (1.01–2.85, 0.048)
Hypertension	1.11 (0.74–1.66, 0.619)
Smoking	1.54 (1.03–2.28, 0.032)
Hepatocellular carcinoma	1.06 (0.68–1.63, 0.808)
MELD-Na	1.01 (0.99–1.03, 0.310)
Hepatic artery thrombosis	1.16 (0.34–3.93, 0.810)
Acute rejection	2.19 (1.42–3.36, < 0.0001)

Abbreviation: MELD-Na = model for end-stage liver disease sodium.

^∗^Test for assumption of proportional hazards was done using Schoenfeld's residuals: *p* value = 0.72 for patient survival models and 0.73 for graft survival models.

## Data Availability

The data that support the findings of this study are available on request from the corresponding author. The data are not publicly available due to privacy or ethical restrictions.

## Nomenclature

ALD: Alcoholic liver disease
BMI: Body mass index
CAD: Coronary artery disease
CT: Computerized tomography
DBD: Donation after brain stem death
DCD: Donation after circulatory death
DDLT: Deceased donor liver transplantation
ESLD: End-stage liver disease
HAT: Hepatic artery thrombosis
HBV: Hepatitis B virus
HCC: Hepatocellular carcinoma
HCV: Hepatitis C virus
HD: Hemodialysis
HRS: Hepatorenal syndrome
ICU: Intensive care unit
INR: International normalized ratio
IQR: Interquartile range
IVC: Inferior vena cava
LDLT: Live donor liver transplantation
LT: Liver transplantation
MDT: Multidisciplinary meeting
MELD: Model of end-stage liver disease
MRI: Magnetic resonance imaging
NAFLD: Nonalcoholic fatty liver disease
OG: Older adult group
OLT: Orthotopic liver transplantation
PBC: Primary biliary cholangitis
PNF: Primary nonfunction
PET: Positron emission tomography
PSC: Primary sclerosing cholangitis
PTLD: Post-transplant lymphoproliferative disorder
PVT: Portal vein thrombosis
SD: Standard deviation
TIPS: Transjugular intrahepatic portosystemic shunt
YG: Younger group
